# Syntheses and crystal structures of 4-(4-nitro­phen­yl)piperazin-1-ium benzoate monohydrate and 4-(4-nitro­phen­yl)piperazin-1-ium 2-carb­oxy-4,6-di­nitro­phenolate

**DOI:** 10.1107/S2056989022007472

**Published:** 2022-07-26

**Authors:** Holehundi J. Shankara Prasad, Devaraju, Vinaya, Hemmige S. Yathirajan, Sean R. Parkin, Christopher Glidewell

**Affiliations:** a Department of Chemistry, Yuvaraja’s College, University of Mysore, Mysore-570 005, India; b Department of Studies in Chemistry, University of Mysore, Manasagangotri, Mysore-570 006, India; c Department of Chemistry, University of Kentucky, Lexington, KY 40506, USA; d School of Chemistry, University of St Andrews, St Andrews, Fife KY16 9ST, UK; University of Aberdeen, Scotland

**Keywords:** piperazine, synthesis, crystal structure, mol­ecular structure, hydrogen bonding, supra­molecular assembly

## Abstract

Crystal structures are reported for two mol­ecular salts containing the 4-(4-nitro­phen­yl)piperazin-1-ium cation. Co-crystallization from methanol/ethyl acetate solution of *N*-(4-nitro­phen­yl)piperazine with benzoic acid gives the benzoate salt, which crystallizes as a monohydrate, C_10_H_14_N_3_O_2_·C_7_H_5_O_2_·H_2_O, (I), and similar co-crystallization with 3,5-di­nitro­salicylic acid yields the 2-carb­oxy-4,6-di­nitro­phenolate salt, C_10_H_14_N_3_O_2_·C_7_H_3_N_2_O_7_, (II). In the structure of (I), a combination of O—H⋯O, N—H⋯O and C—H⋯O hydrogen bonds links the components into sheets, while in the structure of (II), the supra­molecular assembly, generated by hydrogen bonds of the same types as in (I), is three dimensional. Comparisons are made with the structures of some related compounds.

## Abstract 2

Two new salts of the 4-(4-nitro­phen­yl)piperazin-1-ium cation have been prepared by co-crystallization with aromatic carb­oxy­lic acids. The supra­molecular assembly in the benzoate salt, which crystallizes as a mono-hydrate, is two dimensional, while that in the 2-carb­oxy-4,6-di­nitro­phenolate salt is three dimensional.

## 1. Chemical context

Piperazines and substituted piperazines are important pharmacophores, which can be found in many biologically active compounds (Berkheij, 2005 ▸) such as anti­fungal (Upadhayaya *et al.*, 2004 ▸), anti-bacterial, anti-malarial and anti-psychotic agents (Chaudhary *et al.*, 2006 ▸). Both the general pharmacological and specific anti­microbial activities of piperazine derivatives have been reviewed in recent years (Elliott, 2011 ▸; Kharb *et al.*, 2012 ▸). Among specific examples of piperazine derivatives, *N*-(4-nitro­phen­yl)piperazine has found use in the control of potassium channels (Lu, 2007 ▸). The crystal structures of a number of 4-(4-nitro­phen­yl)piperazin-1-ium salts have been reported (Lu, 2007 ▸; Mahesha *et al.*, 2022 ▸), and here we report the mol­ecular and supra­molecular structures of two further representatives of this family of salts, namely 4-(4-nitro­phen­yl)piperazin-1-ium benzoate monohydrate, C_10_H_14_N_3_O_2_·C_7_H_5_O_2_·H_2_O, (I), and 4-(4-nitro­phen­yl)pip­erazin-1-ium 2-carb­oxy-4,6-di­nitro­phenolate, C_10_H_14_N_3_O_2_·C_7_H_3_N_2_O_7_, (II).

## 2. Structural commentary

**Figure 1:**
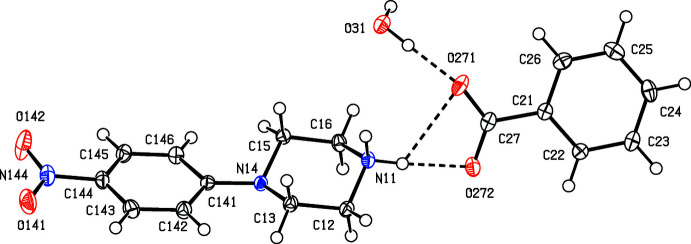
The mol­ecular structure of (I), showing hydrogen bonds (drawn as dashed lines) within the selected asymmetric unit. Displacement ellipsoids are drawn at the 50% probability level.

In each of compounds (I) and (II) (Figs. 1 ▸ and 2 ▸), the piperazine ring adopts a chair conformation, with the ring-puckering angle θ (Cremer & Pople, 1975 ▸) calculated for the atom sequence (N11/C12/C13/N14/C15/C16) close to the ideal value of zero (Boeyens, 1978 ▸): θ = 6.42 (11) for (I) and 8.75 (11)° for (II). However, in (I), the nitro­phenyl substituent occupies an equatorial site, whereas in (II) this substituent occupies an axial site. In each compound, the *N*-nitro­phenyl unit shows the pattern of distances typical of 4-nitro­aniline derivatives, namely both C—N distances are short for their types (Allen *et al.*, 1987 ▸), while the nitro N—O distances are long for their type. In addition, the distances C141—C142 and C141—C146 lie in the range 1.4049 (16) to 1.4132 (15) Å whereas the remaining C—C distances for this ring are smaller, falling in the range 1.3764 (17) to 1.3881 (15) Å. These variations are most simply inter­preted in terms of some 1,4-quinonoid type bond fixation, moderated by the high electronegativity of the nitro group, generally regarded as similar to that of a fluoro substituent (Huheey, 1966 ▸; Mullay, 1985 ▸). 
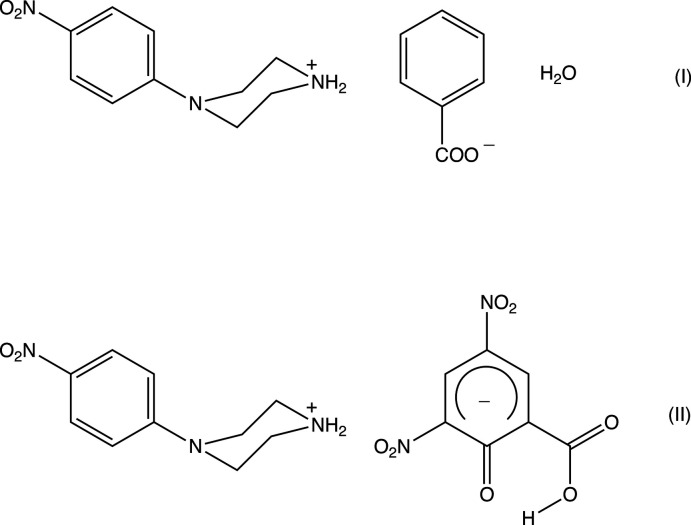


In the anion of compound (II), the C21—O21 distance, 1.2788 (13) Å is more typical of those in ketones than those in phenols (Allen *et al.*, 1987 ▸); the distances C21—C22 and C21—C26, 1.4394 (15) and 1.4340 (15) Å are longer than the remaining C—C distances in the ring, which are in the range 1.3747 (15) to 1.3869 (15). These observations, taken together, indicate that the negative charge in this anion is delocalized over atoms C22–C26 rather than being localized on atom O21 (see Scheme[Chem scheme1]).

## 3. Supra­molecular features

In each of compounds (I) and (II), the supra­molecular assembly involves a combination of O—H⋯O, N—H⋯O and C—H⋯O hydrogen bonds, augmented in the case of (I) by a single C—H⋯π(arene) hydrogen bond: however, aromatic π–π stacking inter­actions are absent from both structures.

The supra­molecular assembly in (I) is di-periodic and the formation of the sheet structure is readily analysed in terms of two mono-periodic sub-structures (Ferguson *et al.*, 1998*a* ▸,*b* ▸; Gregson *et al.*, 2000 ▸). Within the selected asymmetric unit for (I) (Fig. 1 ▸), the ionic components are linked by an asymmetric bifurcated (three-centre) N—H⋯(O,O) hydrogen bond (Table 1 ▸), while the water mol­ecule is linked to the anion by an O—H⋯O hydrogen bond. In one of the two sub-structures, a combination of one two-centre N—H⋯O hydrogen bond and a second O—H⋯O hydrogen bond links these three-component aggregates (Fig. 1 ▸) into a chain of rings running parallel to the [100] direction (Fig. 3 ▸) in which there are two different types of 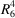 (12) ring (Bernstein *et al.*, 1995 ▸), centred at (*n*, 0.5, 0.5) and (*n* + 0.5, 0.5, 0.5), respectively, where *n* represents an integer in each case. The second sub-structure, which includes the C—H⋯O hydrogen bond (Table 1 ▸, Fig. 4 ▸), takes the form of another chain of rings in which 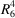 (12) rings centred at (*n* + 0.5, *n* + 0.5, 0.5) alternate with 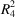 (10) rings centred at (*n*, *n*, 0.5), where *n* again represents an integer, so forming a chain of rings running parallel to the [110] direction (Fig. 4 ▸). The combination of chains along [100] and [110] generates a sheet structure lying parallel to (001). The single C—H⋯π(arene) hydrogen bond (Table 1 ▸) lies within this sheet, and so has no influence on the dimensionality of the assembly.

**Figure 2:**
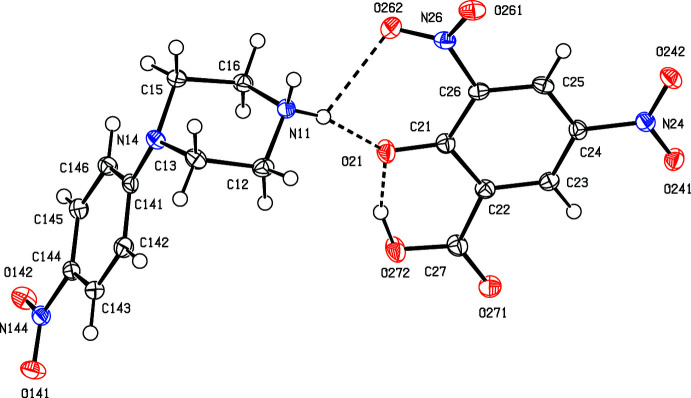
The mol­ecular structure of (II), showing hydrogen bonds (drawn as dashed lines) within the selected asymmetric unit. Displacement ellipsoids are drawn at the 50% probability level.

**Figure 3:**
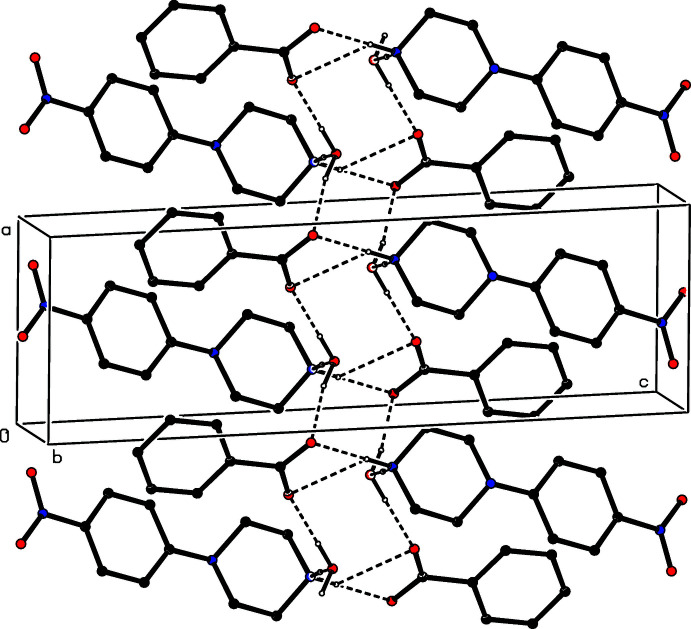
Part of the crystal structure of compound (I) showing the formation of a chain of hydrogen-bonded rings running parallel to the [100] direction. Hydrogen bonds are drawn as dashed lines and, for the sake of clarity, the H atoms bonded to C atoms have all been omitted.

**Figure 4:**
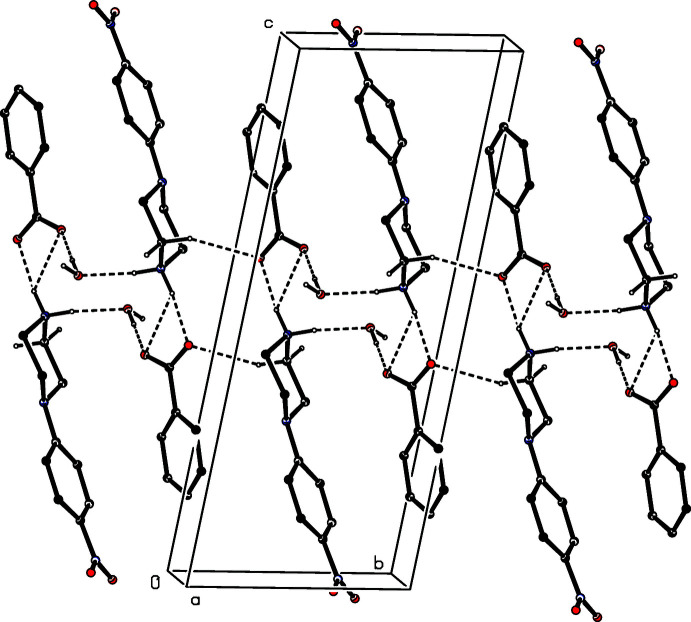
Part of the crystal structure of compound (I) showing the formation of a chain of hydrogen-bonded rings running parallel to the [110] direction. Hydrogen bonds are drawn as dashed lines and, for the sake of clarity, the H atoms bonded to those C atoms that are not involved in the motif shown have been omitted.

**Figure 5:**
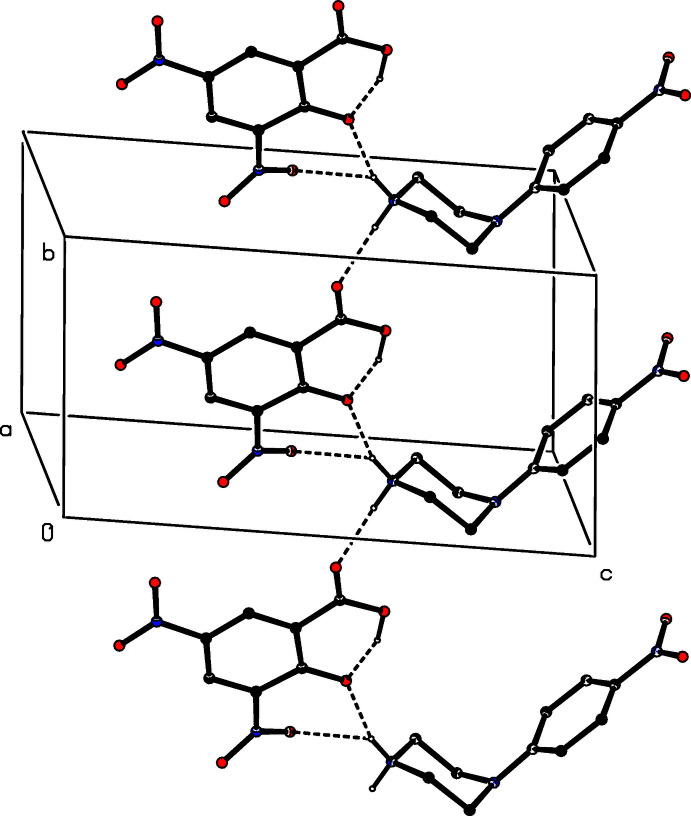
Part of the crystal structure of compound (II), showing the formation of a hydrogen-bonded chain of rings running parallel to [010]. Hydrogen bonds are drawn as dashed lines and, for the sake of clarity, the H atoms bonded to C atoms have all been omitted.

**Figure 6:**
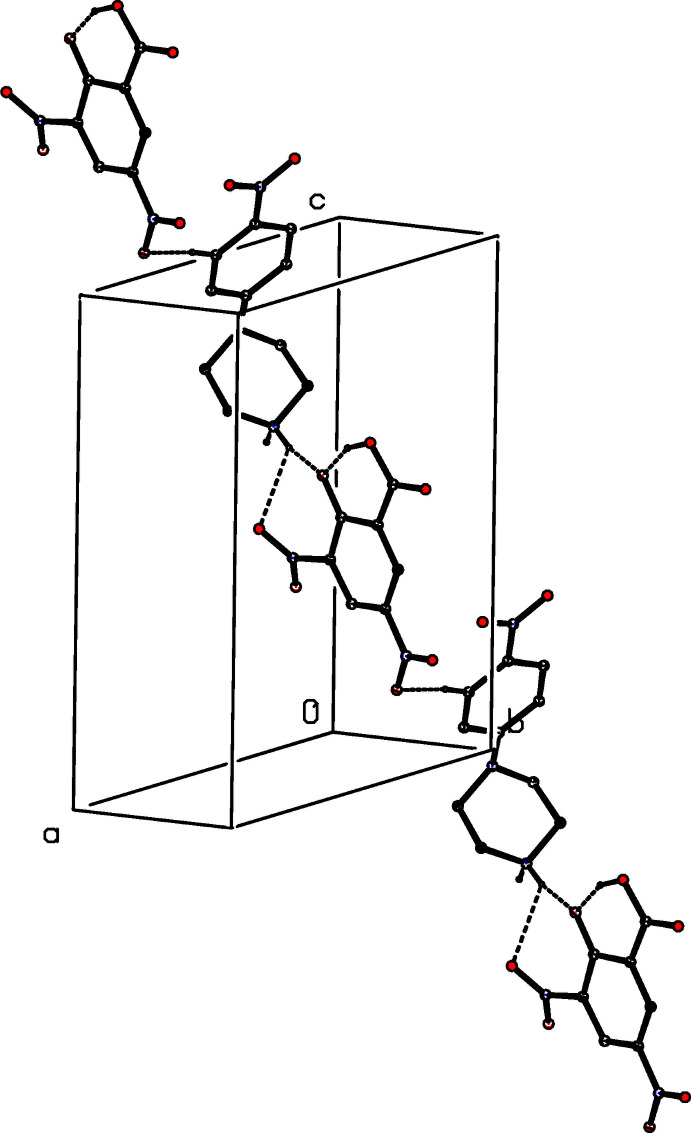
Part of the crystal structure of compound (II) showing the formation of a chain of hydrogen-bonded rings running parallel to the [101] direction. Hydrogen bonds are drawn as dashed lines and, for the sake of clarity, the H atoms bonded to those C atoms that are not involved in the motif shown have been omitted.

**Figure 7:**
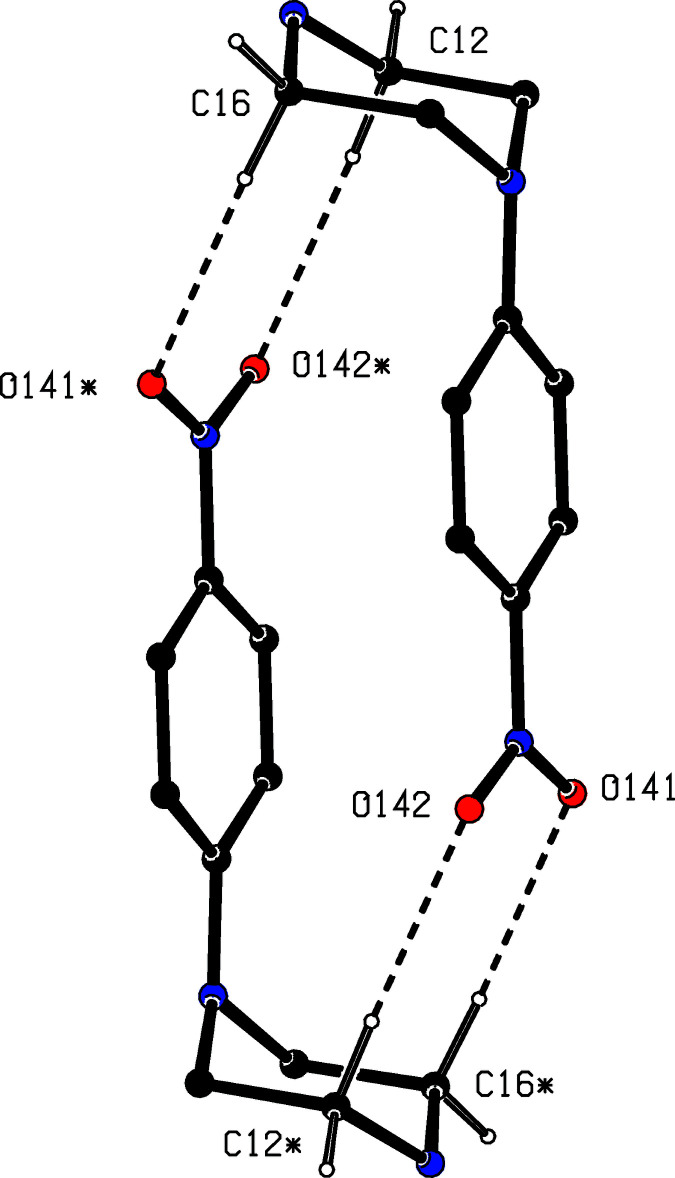
Part of the crystal structure of compound (II) showing the linkage of an inversion-related pair of cations by two independent C—H⋯O hydrogen bonds, drawn as dashed lines. For the sake of clarity, the anions, the H atoms bonded to those C atoms that are not involved in the motif shown, and the unit-cell outline have been omitted. The atoms marked with an asterisk (*) are at the symmetry position (1 − *x*, 1 − *y*, 2 − *z*).

**Figure 8:**
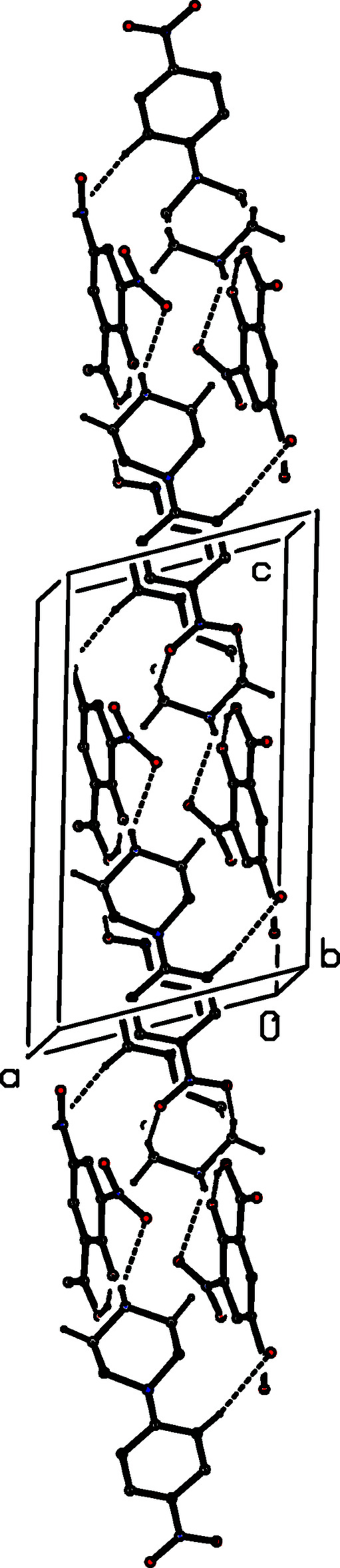
Part of the crystal structure of compound (II) showing the formation of a chain of hydrogen-bonded rings running parallel to the [001] direction. Hydrogen bonds are drawn as dashed lines and, for the sake of clarity, the H atoms bonded to those C atoms that are not involved in the motif shown have been omitted.

The supra­molecular assembly for compound (II), by contrast, is tri-periodic (three dimensional) and, as for (I), the formation of the framework is readily analysed in terms of simple sub-structures. Within the selected asymmetric unit (Fig. 2 ▸), there is an intra­molecular O—H⋯O hydrogen bond in the anion, and the hydroxyl H atom plays no part in the supra­molecular assembly. The two independent components are linked by a very asymmetric bifurcated N—H⋯(O,O) hydrogen bond (Table 2 ▸), and a two-centre N—H⋯O hydrogen bond links these ion pairs into a chain of rings running parallel to the [010] direction (Fig. 5 ▸). There are four C—H⋯O hydrogen bonds in the structure of (II) and that involving atom C145 (Table 2 ▸) links the ion pairs into a second chain, this time running parallel to the [101] direction (Fig. 6 ▸). The two C—H⋯O hydrogen bonds involving atoms C12 and C16 link inversion-related pairs of cations into a centrosymmetric motif containing 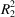 (8) rings (Fig. 7 ▸), and the aggregates of this type are further linked by the final C—H⋯O hydrogen bond, that involves atom C146, to form a complex chain of rings running parallel to the [001] direction (Fig. 8 ▸). The combination of hydrogen-bonded chains parallel to [010], [001] and [101] generates a three-dimensional network. We also note a fairly short nitro–nitro contact, 2.823 (4) Å, between atom O142 at (*x*, *y*, *z*) and atom N24 at (1 + *x*, *y*, 1 + *z*): this probably represents a dipolar attraction between negatively charged O and positively charged N atoms.

## 4. Database survey

The first structure report on a salt of *N*-(4-nitro­phen­yl)piperazine concerned the chloride salt, which crystallizes as a monohydrate (Lu, 2007 ▸); despite the presence of hydrogen bonds of N—H⋯O, N—H⋯Cl and O—H⋯Cl types, the supra­molecular assembly is only mono-periodic. The structures of six salts of *N*-(4-nitro­phen­yl)piperazine with aromatic carb­oxy­lic acids have recently been reported (Mahesha *et al.*, 2022 ▸): in all but one of these, the supra­molecular assembly is mono-periodic, although it is di-periodic in the 4-eth­oxy­benzoate salt. This may be contrasted with the triperiodic assembly found here for compound (II).

In addition, we note that structures have been reported for a wide variety of salts derived from *N*-(4-fluoro­phen­yl)piperazine (Harish Chinthal, Yathirajan, Archana *et al.*, 2020 ▸; Harish Chinthal, Yathirajan, Kavitha *et al.*, 2020 ▸), and from *N*-(4-meth­oxy­phen­yl)piperazine (Kiran Kumar *et al.*, 2019 ▸, 2020 ▸). Finally, the structure of 4-(2-meth­oxy­phen­yl)piperazin-1-ium 3,5-di­nitro­salicylate has been reported, but without any description of discussion of the geometry of the anion (Subha *et al.*, 2022 ▸).

## 5. Synthesis and crystallization

For the preparation of compounds (I) and (II), a solution of *N*-(4-nitro­phen­yl)piperazine (100 mg, 0.483 mmol) in methanol (10 ml) was mixed with a solution of either benzoic acid (59 mg, 0.483 mmol) for (I) or 3,5-di­nitro­salicylic acid (110 mg, 0.483 mmol) for (II) in methanol/ethyl acetate (1:1 *v*/*v*, 20 ml). The solutions of the base and the corresponding acid were mixed, stirred at ambient temperature for 15 min, and then set aside to crystallize at ambient temperature and in the presence of air. After one week, crystals suitable for single-crystal X-ray diffraction were collected by filtration and dried in air: compound (I), pale yellow, m.p. 410–413 K; compound (II), orange, m.p. 446–448 K.

## 6. Refinement

Crystal data, data collection and refinement details are summarized in Table 3 ▸. All H atoms were located in difference maps. The H atoms bonded to C atoms were then treated as riding atoms in geometrically idealized positions with C—H distances of 0.95 Å (aromatic) or 0.99 Å (CH_2_), and with *U*_iso_(H) = 1.2*U*_eq_(C). For the H atoms bonded to N or O atoms, the atomic coordinates were refined with *U*_iso_(H) = 1.2*U*_eq_(*N*) or 1.5*U*_eq_(O), giving the N—H and O—H distances shown in Tables 1 ▸ and 2 ▸.

## Supplementary Material

Crystal structure: contains datablock(s) global, I, II. DOI: 10.1107/S2056989022007472/hb8030sup1.cif

Structure factors: contains datablock(s) I. DOI: 10.1107/S2056989022007472/hb8030Isup2.hkl

Structure factors: contains datablock(s) II. DOI: 10.1107/S2056989022007472/hb8030IIsup3.hkl

Supporting information file. DOI: 10.1107/S2056989022007472/hb8030Isup4.cml

Supporting information file. DOI: 10.1107/S2056989022007472/hb8030IIsup5.cml

CCDC references: 2191691, 2191690

Additional supporting information:  crystallographic information; 3D view; checkCIF report

## Figures and Tables

**Table 1:** Hydrogen-bond geometry (Å, °) for (I) *Cg*1 is the centroid of the C21–C26 ring.

*D*—H⋯*A*	*D*—H	H⋯*A*	*D*⋯*A*	*D*—H⋯*A*
N11—H11⋯O271	0.926 (14)	2.564 (14)	3.1009 (13)	117.4 (10)
N11—H11⋯O272	0.926 (14)	1.857 (14)	2.7781 (12)	172.9 (13)
N11—H12⋯O31^i^	0.920 (15)	1.884 (15)	2.7965 (14)	171.0 (12)
O31—H31⋯O271	0.892 (18)	1.757 (18)	2.6486 (13)	179 (3)
O31—H32⋯O272^ii^	0.908 (17)	1.862 (17)	2.7581 (12)	168.8 (16)
C12—H12*B*⋯O272^iii^	0.99	2.45	3.3751 (15)	156
C146—H146⋯*Cg*1^iv^	0.95	2.67	3.4363 (13)	138

Symmetry codes: (i) 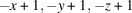 ; (ii) 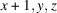 ; (iii) 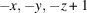 ; (iv) 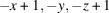 .

**Table 2:** Hydrogen-bond geometry (Å, °) for (II)

*D*—H⋯*A*	*D*—H	H⋯*A*	*D*⋯*A*	*D*—H⋯*A*
N11—H11⋯O21	0.894 (14)	1.869 (14)	2.7356 (12)	162.8 (13)
N11—H11⋯O262	0.894 (14)	2.396 (14)	2.8937 (13)	115.4 (11)
N11—H12⋯O271^i^	0.910 (14)	1.874 (14)	2.7668 (12)	166.2 (12)
O272—H272⋯O21	1.000 (17)	1.549 (17)	2.5020 (12)	157.4 (15)
C12—H12*B*⋯O142^ii^	0.99	2.41	3.3921 (14)	173
C16—H16*A*⋯O141^ii^	0.99	2.54	3.4906 (14)	161
C145—H145⋯O242^iii^	0.95	2.46	3.3927 (15)	168
C146—H146⋯O241^iv^	0.95	2.55	3.4227 (15)	153

Symmetry codes: (i) 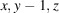 ; (ii) 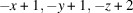 ; (iii) 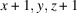 ; (iv) 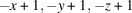 .

**Table 3:** Experimental details

	(I)	(II)
Crystal data		
Chemical formula	C_10_H_14_N_3_O_2_^+^·C_7_H_5_O_2_^−^·H_2_O	C_10_H_14_N_3_O_2_^+^·C_7_H_3_N_2_O_7_^−^
*M*_r_	347.37	435.35
Crystal system, space group	Triclinic, *P* 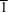	Triclinic, *P* 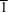
Temperature (K)	90	90
*a*, *b*, *c* (Å)	6.0768 (3), 7.4427 (4), 18.4737 (9)	7.9599 (4), 8.5391 (4), 14.2227 (5)
α, β, γ (°)	78.894 (2), 85.870 (3), 83.668 (2)	90.426 (2), 105.273 (1), 98.538 (2)
*V* (Å^3^)	813.77 (7)	921.15 (7)
*Z*	2	2
Radiation type	Mo *K*α	Mo *K*α
μ (mm^−1^)	0.11	0.13
Crystal size (mm)	0.24 × 0.22 × 0.17	0.22 × 0.18 × 0.12

Data collection		
Diffractometer	Bruker D8 Venture	Bruker D8 Venture
Absorption correction	Multi-scan (*SADABS*; Krause *et al.*, 2015 ▸)	Multi-scan (*SADABS*; Krause *et al.*, 2015 ▸)
*T*_min_, *T*_max_	0.912, 0.971	0.919, 0.971
No. of measured, independent and observed [*I* > 2σ(*I*)] reflections	27142, 3737, 3164	38287, 4212, 3662
*R*_int_	0.066	0.043
(sin θ/λ)_max_ (Å^−1^)	0.651	0.650

Refinement		
*R*[*F* ^2^ > 2σ(*F* ^2^)], *wR*(*F* ^2^), *S*	0.036, 0.091, 1.04	0.029, 0.077, 1.04
No. of reflections	3737	4212
No. of parameters	238	289
H-atom treatment	H atoms treated by a mixture of independent and constrained refinement	H atoms treated by a mixture of independent and constrained refinement
Δρ_max_, Δρ_min_ (e Å^−3^)	0.25, −0.20	0.28, −0.18

Computer programs: *APEX3* (Bruker, 2016 ▸), *SHELXT2014/5* (Sheldrick, 2015*a* ▸), *SHELXL2014* (Sheldrick, 2015*b* ▸) and *PLATON* (Spek, 2020 ▸).

## supplementary crystallographic information

### 4-(4-Nitrophenyl)piperazin-1-ium benzoate monohydrate (I) Crystal data

**Table:**

C_10_H_14_N_3_O_2_^+^·C_7_H_5_O_2_^−^·H_2_O	*Z* = 2
*M**_r_* = 347.37	*F*(000) = 368
Triclinic, *P*1	*D*_x_ = 1.418 Mg m^−^^3^
*a* = 6.0768 (3) Å	Mo *K*α radiation, λ = 0.71073 Å
*b* = 7.4427 (4) Å	Cell parameters from 3737 reflections
*c* = 18.4737 (9) Å	θ = 2.3–27.6°
α = 78.894 (2)°	µ = 0.11 mm^−^^1^
β = 85.870 (3)°	*T* = 90 K
γ = 83.668 (2)°	Block, pale yellow
*V* = 813.77 (7) Å^3^	0.24 × 0.22 × 0.17 mm

### 4-(4-Nitrophenyl)piperazin-1-ium benzoate monohydrate (I) Data collection

**Table:**

Bruker D8 Venture diffractometer	3737 independent reflections
Radiation source: microsource	3164 reflections with *I* > 2σ(*I*)
Multilayer mirror monochromator	*R*_int_ = 0.066
φ and ω scans	θ_max_ = 27.6°, θ_min_ = 2.3°
Absorption correction: multi-scan (SADABS; Krause *et al.*, 2015)	*h* = −7→7
*T*_min_ = 0.912, *T*_max_ = 0.971	*k* = −9→9
27142 measured reflections	*l* = −23→23

### 4-(4-Nitrophenyl)piperazin-1-ium benzoate monohydrate (I) Refinement

**Table:**

Refinement on *F*^2^	Primary atom site location: dual
Least-squares matrix: full	Hydrogen site location: mixed
*R*[*F*^2^ > 2σ(*F*^2^)] = 0.036	H atoms treated by a mixture of independent and constrained refinement
*wR*(*F*^2^) = 0.091	*w* = 1/[σ^2^(*F*_o_^2^) + (0.030*P*)^2^ + 0.2902*P*] where *P* = (*F*_o_^2^ + 2*F*_c_^2^)/3
*S* = 1.04	(Δ/σ)_max_ = 0.001
3737 reflections	Δρ_max_ = 0.25 e Å^−^^3^
238 parameters	Δρ_min_ = −0.20 e Å^−^^3^
0 restraints	

### 4-(4-Nitrophenyl)piperazin-1-ium benzoate monohydrate (I) Special details

**Table:**

Geometry. All esds (except the esd in the dihedral angle between two l.s. planes) are estimated using the full covariance matrix. The cell esds are taken into account individually in the estimation of esds in distances, angles and torsion angles; correlations between esds in cell parameters are only used when they are defined by crystal symmetry. An approximate (isotropic) treatment of cell esds is used for estimating esds involving l.s. planes.

### 4-(4-Nitrophenyl)piperazin-1-ium benzoate monohydrate (I) Fractional atomic coordinates and isotropic or equivalent isotropic displacement parameters (Å^2^)

**Table:**

	*x*	*y*	*z*	*U*_iso_*/*U*_eq_	
N11	0.22281 (16)	0.29238 (14)	0.44679 (5)	0.0163 (2)	
H11	0.172 (2)	0.2234 (19)	0.4908 (8)	0.020*	
H12	0.255 (2)	0.403 (2)	0.4565 (7)	0.020*	
C12	0.04640 (18)	0.33021 (16)	0.39257 (6)	0.0175 (2)	
H12A	−0.0854	0.3983	0.4129	0.021*	
H12B	0.0020	0.2124	0.3839	0.021*	
C13	0.12736 (18)	0.44242 (16)	0.32002 (6)	0.0172 (2)	
H13A	0.0112	0.4575	0.2838	0.021*	
H13B	0.1524	0.5664	0.3278	0.021*	
N14	0.33332 (15)	0.35614 (13)	0.28973 (5)	0.0147 (2)	
C15	0.50502 (18)	0.30670 (16)	0.34411 (6)	0.0163 (2)	
H15A	0.5561	0.4205	0.3546	0.020*	
H15B	0.6336	0.2365	0.3230	0.020*	
C16	0.42143 (19)	0.19258 (16)	0.41561 (6)	0.0177 (2)	
H16A	0.3830	0.0734	0.4063	0.021*	
H16B	0.5394	0.1671	0.4515	0.021*	
C141	0.40488 (18)	0.44038 (15)	0.21853 (6)	0.0149 (2)	
C142	0.26572 (19)	0.57118 (16)	0.17307 (6)	0.0199 (2)	
H142	0.1188	0.6040	0.1906	0.024*	
C143	0.3394 (2)	0.65311 (16)	0.10304 (6)	0.0213 (3)	
H143	0.2432	0.7399	0.0725	0.026*	
C144	0.5535 (2)	0.60750 (16)	0.07820 (6)	0.0189 (2)	
C145	0.6944 (2)	0.47632 (16)	0.12051 (6)	0.0199 (2)	
H145	0.8406	0.4442	0.1021	0.024*	
C146	0.61972 (19)	0.39295 (16)	0.18972 (6)	0.0178 (2)	
H146	0.7152	0.3015	0.2186	0.021*	
N144	0.63672 (18)	0.70469 (14)	0.00703 (6)	0.0242 (2)	
O141	0.50758 (17)	0.81136 (14)	−0.03222 (5)	0.0352 (2)	
O142	0.83458 (17)	0.67621 (15)	−0.01004 (6)	0.0421 (3)	
C21	0.13555 (18)	0.09842 (14)	0.71032 (6)	0.0156 (2)	
C22	−0.05414 (19)	0.01147 (15)	0.73666 (6)	0.0170 (2)	
H22	−0.1565	−0.0072	0.7030	0.020*	
C23	−0.0937 (2)	−0.04784 (16)	0.81206 (6)	0.0207 (2)	
H23	−0.2239	−0.1058	0.8299	0.025*	
C24	0.0564 (2)	−0.02246 (16)	0.86119 (6)	0.0229 (3)	
H24	0.0297	−0.0636	0.9127	0.027*	
C25	0.2458 (2)	0.06303 (16)	0.83528 (7)	0.0236 (3)	
H25	0.3494	0.0793	0.8691	0.028*	
C26	0.2842 (2)	0.12465 (16)	0.76032 (7)	0.0203 (2)	
H26	0.4127	0.1852	0.7429	0.024*	
C27	0.18134 (19)	0.16532 (15)	0.62878 (6)	0.0179 (2)	
O271	0.32916 (15)	0.27141 (13)	0.61005 (5)	0.0293 (2)	
O272	0.06693 (14)	0.11185 (11)	0.58360 (4)	0.02171 (19)	
O31	0.70609 (15)	0.35347 (12)	0.53628 (5)	0.02152 (19)	
H31	0.579 (3)	0.325 (2)	0.5608 (9)	0.032*	
H32	0.813 (3)	0.268 (2)	0.5570 (9)	0.032*	

### 4-(4-Nitrophenyl)piperazin-1-ium benzoate monohydrate (I) Atomic displacement parameters (Å^2^)

**Table:**

	*U*^11^	*U*^22^	*U*^33^	*U*^12^	*U*^13^	*U*^23^
N11	0.0197 (5)	0.0150 (5)	0.0136 (5)	−0.0029 (4)	0.0017 (4)	−0.0017 (4)
C12	0.0152 (5)	0.0199 (6)	0.0168 (5)	−0.0021 (4)	0.0012 (4)	−0.0027 (4)
C13	0.0147 (5)	0.0189 (5)	0.0165 (5)	0.0002 (4)	0.0009 (4)	−0.0011 (4)
N14	0.0129 (4)	0.0170 (5)	0.0133 (4)	−0.0004 (4)	0.0003 (3)	−0.0016 (3)
C15	0.0142 (5)	0.0186 (5)	0.0153 (5)	0.0005 (4)	−0.0002 (4)	−0.0023 (4)
C16	0.0189 (6)	0.0174 (5)	0.0154 (5)	0.0014 (4)	−0.0004 (4)	−0.0015 (4)
C141	0.0166 (5)	0.0138 (5)	0.0149 (5)	−0.0035 (4)	0.0003 (4)	−0.0038 (4)
C142	0.0173 (6)	0.0229 (6)	0.0178 (6)	0.0004 (5)	0.0000 (4)	−0.0011 (4)
C143	0.0238 (6)	0.0203 (6)	0.0181 (6)	0.0008 (5)	−0.0032 (5)	−0.0002 (4)
C144	0.0267 (6)	0.0166 (5)	0.0138 (5)	−0.0061 (5)	0.0018 (4)	−0.0023 (4)
C145	0.0203 (6)	0.0205 (6)	0.0186 (6)	−0.0023 (5)	0.0036 (4)	−0.0048 (4)
C146	0.0181 (6)	0.0167 (5)	0.0174 (5)	0.0009 (4)	0.0003 (4)	−0.0020 (4)
N144	0.0324 (6)	0.0222 (5)	0.0170 (5)	−0.0051 (4)	0.0035 (4)	−0.0018 (4)
O141	0.0423 (6)	0.0372 (6)	0.0203 (5)	−0.0024 (5)	−0.0027 (4)	0.0089 (4)
O142	0.0364 (6)	0.0463 (6)	0.0329 (5)	0.0013 (5)	0.0165 (4)	0.0092 (5)
C21	0.0167 (5)	0.0110 (5)	0.0180 (5)	0.0009 (4)	0.0008 (4)	−0.0019 (4)
C22	0.0169 (5)	0.0158 (5)	0.0186 (5)	−0.0011 (4)	0.0002 (4)	−0.0043 (4)
C23	0.0226 (6)	0.0172 (6)	0.0209 (6)	−0.0024 (5)	0.0060 (5)	−0.0026 (4)
C24	0.0348 (7)	0.0164 (6)	0.0158 (5)	0.0018 (5)	0.0008 (5)	−0.0021 (4)
C25	0.0302 (7)	0.0180 (6)	0.0236 (6)	0.0003 (5)	−0.0100 (5)	−0.0047 (5)
C26	0.0190 (6)	0.0142 (5)	0.0271 (6)	−0.0019 (4)	−0.0013 (5)	−0.0024 (4)
C27	0.0171 (5)	0.0130 (5)	0.0211 (6)	0.0017 (4)	0.0036 (4)	−0.0004 (4)
O271	0.0260 (5)	0.0318 (5)	0.0282 (5)	−0.0127 (4)	0.0070 (4)	0.0012 (4)
O272	0.0275 (5)	0.0213 (4)	0.0157 (4)	−0.0036 (3)	0.0013 (3)	−0.0020 (3)
O31	0.0203 (4)	0.0214 (4)	0.0228 (4)	−0.0045 (4)	0.0042 (3)	−0.0044 (3)

### 4-(4-Nitrophenyl)piperazin-1-ium benzoate monohydrate (I) Geometric parameters (Å, º)

**Table:**

N11—C16	1.4857 (14)	C144—C145	1.3841 (17)
N11—C12	1.4870 (14)	C144—N144	1.4581 (14)
N11—H11	0.925 (14)	C145—C146	1.3780 (16)
N11—H12	0.921 (15)	C145—H145	0.9500
C12—C13	1.5167 (15)	C146—H146	0.9500
C12—H12A	0.9900	N144—O141	1.2226 (14)
C12—H12B	0.9900	N144—O142	1.2268 (14)
C13—N14	1.4671 (14)	C21—C26	1.3911 (16)
C13—H13A	0.9900	C21—C22	1.3953 (16)
C13—H13B	0.9900	C21—C27	1.5084 (15)
N14—C141	1.4044 (14)	C22—C23	1.3896 (16)
N14—C15	1.4688 (14)	C22—H22	0.9500
C15—C16	1.5134 (15)	C23—C24	1.3840 (18)
C15—H15A	0.9900	C23—H23	0.9500
C15—H15B	0.9900	C24—C25	1.3869 (18)
C16—H16A	0.9900	C24—H24	0.9500
C16—H16B	0.9900	C25—C26	1.3838 (17)
C141—C142	1.4049 (16)	C25—H25	0.9500
C141—C146	1.4081 (16)	C26—H26	0.9500
C142—C143	1.3853 (16)	C27—O271	1.2476 (14)
C142—H142	0.9500	C27—O272	1.2692 (14)
C143—C144	1.3764 (17)	O31—H31	0.893 (17)
C143—H143	0.9500	O31—H32	0.909 (17)
			
C16—N11—C12	109.33 (9)	C141—C142—H142	119.5
C16—N11—H11	110.2 (8)	C144—C143—C142	119.36 (11)
C12—N11—H11	110.3 (8)	C144—C143—H143	120.3
C16—N11—H12	111.3 (8)	C142—C143—H143	120.3
C12—N11—H12	108.0 (8)	C143—C144—C145	121.48 (11)
H11—N11—H12	107.6 (12)	C143—C144—N144	119.32 (11)
N11—C12—C13	110.80 (9)	C145—C144—N144	119.16 (11)
N11—C12—H12A	109.5	C146—C145—C144	119.05 (11)
C13—C12—H12A	109.5	C146—C145—H145	120.5
N11—C12—H12B	109.5	C144—C145—H145	120.5
C13—C12—H12B	109.5	C145—C146—C141	121.42 (10)
H12A—C12—H12B	108.1	C145—C146—H146	119.3
N14—C13—C12	112.32 (9)	C141—C146—H146	119.3
N14—C13—H13A	109.1	O141—N144—O142	123.28 (11)
C12—C13—H13A	109.1	O141—N144—C144	118.78 (11)
N14—C13—H13B	109.1	O142—N144—C144	117.94 (10)
C12—C13—H13B	109.1	C26—C21—C22	119.26 (10)
H13A—C13—H13B	107.9	C26—C21—C27	119.54 (10)
C141—N14—C13	115.67 (9)	C22—C21—C27	121.19 (10)
C141—N14—C15	115.77 (9)	C23—C22—C21	120.20 (11)
C13—N14—C15	112.34 (9)	C23—C22—H22	119.9
N14—C15—C16	112.12 (9)	C21—C22—H22	119.9
N14—C15—H15A	109.2	C24—C23—C22	119.98 (11)
C16—C15—H15A	109.2	C24—C23—H23	120.0
N14—C15—H15B	109.2	C22—C23—H23	120.0
C16—C15—H15B	109.2	C23—C24—C25	120.06 (11)
H15A—C15—H15B	107.9	C23—C24—H24	120.0
N11—C16—C15	110.12 (9)	C25—C24—H24	120.0
N11—C16—H16A	109.6	C26—C25—C24	120.10 (11)
C15—C16—H16A	109.6	C26—C25—H25	119.9
N11—C16—H16B	109.6	C24—C25—H25	119.9
C15—C16—H16B	109.6	C25—C26—C21	120.38 (11)
H16A—C16—H16B	108.2	C25—C26—H26	119.8
N14—C141—C142	121.85 (10)	C21—C26—H26	119.8
N14—C141—C146	120.53 (10)	O271—C27—O272	124.07 (11)
C142—C141—C146	117.62 (10)	O271—C27—C21	117.57 (11)
C143—C142—C141	121.01 (11)	O272—C27—C21	118.35 (10)
C143—C142—H142	119.5	H31—O31—H32	106.4 (14)
			
C16—N11—C12—C13	−58.34 (12)	C144—C145—C146—C141	−0.95 (17)
N11—C12—C13—N14	54.53 (12)	N14—C141—C146—C145	−178.85 (10)
C12—C13—N14—C141	172.83 (9)	C142—C141—C146—C145	2.33 (17)
C12—C13—N14—C15	−51.20 (12)	C143—C144—N144—O141	−7.29 (17)
C141—N14—C15—C16	−171.67 (9)	C145—C144—N144—O141	175.18 (11)
C13—N14—C15—C16	52.40 (12)	C143—C144—N144—O142	171.98 (12)
C12—N11—C16—C15	59.27 (12)	C145—C144—N144—O142	−5.55 (17)
N14—C15—C16—N11	−56.67 (12)	C26—C21—C22—C23	0.09 (16)
C13—N14—C141—C142	−13.70 (15)	C27—C21—C22—C23	−179.53 (10)
C15—N14—C141—C142	−148.15 (11)	C21—C22—C23—C24	−0.69 (17)
C13—N14—C141—C146	167.53 (10)	C22—C23—C24—C25	0.35 (18)
C15—N14—C141—C146	33.07 (14)	C23—C24—C25—C26	0.59 (18)
N14—C141—C142—C143	179.86 (11)	C24—C25—C26—C21	−1.19 (17)
C146—C141—C142—C143	−1.33 (17)	C22—C21—C26—C25	0.85 (17)
C141—C142—C143—C144	−1.02 (18)	C27—C21—C26—C25	−179.52 (10)
C142—C143—C144—C145	2.49 (18)	C26—C21—C27—O271	−12.91 (16)
C142—C143—C144—N144	−174.99 (11)	C22—C21—C27—O271	166.71 (11)
C143—C144—C145—C146	−1.51 (18)	C26—C21—C27—O272	167.61 (10)
N144—C144—C145—C146	175.97 (10)	C22—C21—C27—O272	−12.77 (16)

### 4-(4-Nitrophenyl)piperazin-1-ium benzoate monohydrate (I) Hydrogen-bond geometry (Å, º)

*Cg*1 is the centroid of the C21–C26 ring.

**Table:**

*D*—H···*A*	*D*—H	H···*A*	*D*···*A*	*D*—H···*A*
N11—H11···O271	0.926 (14)	2.564 (14)	3.1009 (13)	117.4 (10)
N11—H11···O272	0.926 (14)	1.857 (14)	2.7781 (12)	172.9 (13)
N11—H12···O31^i^	0.920 (15)	1.884 (15)	2.7965 (14)	171.0 (12)
O31—H31···O271	0.892 (18)	1.757 (18)	2.6486 (13)	179 (3)
O31—H32···O272^ii^	0.908 (17)	1.862 (17)	2.7581 (12)	168.8 (16)
C12—H12*B*···O272^iii^	0.99	2.45	3.3751 (15)	156
C146—H146···*Cg*1^iv^	0.95	2.67	3.4363 (13)	138

Symmetry codes: (i) −*x*+1, −*y*+1, −*z*+1; (ii) *x*+1, *y*, *z*; (iii) −*x*, −*y*, −*z*+1; (iv) −*x*+1, −*y*, −*z*+1.

### 4-(4-Nitrophenyl)piperazin-1-ium 2-carboxy-4,6-dinitrophenolate (II) Crystal data

**Table:**

C_10_H_14_N_3_O_2_^+^·C_7_H_3_N_2_O_7_^−^	*Z* = 2
*M**_r_* = 435.35	*F*(000) = 452
Triclinic, *P*1	*D*_x_ = 1.570 Mg m^−^^3^
*a* = 7.9599 (4) Å	Mo *K*α radiation, λ = 0.71073 Å
*b* = 8.5391 (4) Å	Cell parameters from 4212 reflections
*c* = 14.2227 (5) Å	θ = 2.4–27.5°
α = 90.426 (2)°	µ = 0.13 mm^−^^1^
β = 105.273 (1)°	*T* = 90 K
γ = 98.538 (2)°	Block, orange
*V* = 921.15 (7) Å^3^	0.22 × 0.18 × 0.12 mm

### 4-(4-Nitrophenyl)piperazin-1-ium 2-carboxy-4,6-dinitrophenolate (II) Data collection

**Table:**

Bruker D8 Venture diffractometer	4212 independent reflections
Radiation source: microsource	3662 reflections with *I* > 2σ(*I*)
Multilayer mirror monochromator	*R*_int_ = 0.043
φ and ω scans	θ_max_ = 27.5°, θ_min_ = 2.4°
Absorption correction: multi-scan (SADABS; Krause *et al.*, 2015)	*h* = −10→10
*T*_min_ = 0.919, *T*_max_ = 0.971	*k* = −11→11
38287 measured reflections	*l* = −18→17

### 4-(4-Nitrophenyl)piperazin-1-ium 2-carboxy-4,6-dinitrophenolate (II) Refinement

**Table:**

Refinement on *F*^2^	Primary atom site location: dual
Least-squares matrix: full	Hydrogen site location: mixed
*R*[*F*^2^ > 2σ(*F*^2^)] = 0.029	H atoms treated by a mixture of independent and constrained refinement
*wR*(*F*^2^) = 0.077	*w* = 1/[σ^2^(*F*_o_^2^) + (0.029*P*)^2^ + 0.3527*P*] where *P* = (*F*_o_^2^ + 2*F*_c_^2^)/3
*S* = 1.04	(Δ/σ)_max_ = 0.001
4212 reflections	Δρ_max_ = 0.28 e Å^−^^3^
289 parameters	Δρ_min_ = −0.18 e Å^−^^3^
0 restraints	

### 4-(4-Nitrophenyl)piperazin-1-ium 2-carboxy-4,6-dinitrophenolate (II) Special details

**Table:**

Geometry. All esds (except the esd in the dihedral angle between two l.s. planes) are estimated using the full covariance matrix. The cell esds are taken into account individually in the estimation of esds in distances, angles and torsion angles; correlations between esds in cell parameters are only used when they are defined by crystal symmetry. An approximate (isotropic) treatment of cell esds is used for estimating esds involving l.s. planes.

### 4-(4-Nitrophenyl)piperazin-1-ium 2-carboxy-4,6-dinitrophenolate (II) Fractional atomic coordinates and isotropic or equivalent isotropic displacement parameters (Å^2^)

**Table:**

	*x*	*y*	*z*	*U*_iso_*/*U*_eq_	
N11	0.30883 (12)	0.10386 (11)	0.64405 (7)	0.01693 (19)	
H11	0.2967 (17)	0.1840 (17)	0.6040 (10)	0.020*	
H12	0.2796 (17)	0.0129 (17)	0.6057 (10)	0.020*	
C12	0.17915 (14)	0.10314 (13)	0.70340 (8)	0.0188 (2)	
H12A	0.0579	0.0733	0.6608	0.023*	
H12B	0.1890	0.2107	0.7328	0.023*	
C13	0.21403 (14)	−0.01389 (13)	0.78350 (8)	0.0187 (2)	
H13A	0.1321	−0.0092	0.8248	0.022*	
H13B	0.1931	−0.1229	0.7541	0.022*	
N14	0.39625 (12)	0.02405 (11)	0.84356 (6)	0.01699 (19)	
C15	0.51561 (14)	0.00483 (13)	0.78325 (8)	0.0170 (2)	
H15A	0.4884	−0.1043	0.7536	0.020*	
H15B	0.6388	0.0215	0.8245	0.020*	
C16	0.49616 (14)	0.12299 (13)	0.70345 (8)	0.0172 (2)	
H16A	0.5334	0.2322	0.7330	0.021*	
H16B	0.5729	0.1055	0.6610	0.021*	
C141	0.44287 (14)	0.13710 (12)	0.91966 (7)	0.0156 (2)	
C142	0.31639 (14)	0.18318 (13)	0.96323 (8)	0.0185 (2)	
H142	0.1955	0.1406	0.9370	0.022*	
C143	0.36498 (14)	0.28859 (13)	1.04300 (8)	0.0182 (2)	
H143	0.2783	0.3179	1.0717	0.022*	
C144	0.54137 (14)	0.35176 (12)	1.08126 (7)	0.0161 (2)	
C145	0.66886 (14)	0.31362 (13)	1.03852 (8)	0.0173 (2)	
H145	0.7887	0.3601	1.0640	0.021*	
C146	0.62025 (14)	0.20794 (13)	0.95897 (8)	0.0174 (2)	
H146	0.7078	0.1821	0.9299	0.021*	
N144	0.59245 (12)	0.46290 (11)	1.16455 (6)	0.01728 (19)	
O141	0.47859 (11)	0.49209 (10)	1.20379 (6)	0.02406 (19)	
O142	0.74805 (11)	0.52457 (10)	1.19410 (6)	0.02329 (18)	
C21	0.24532 (14)	0.43648 (13)	0.46951 (8)	0.0171 (2)	
C22	0.20094 (14)	0.59329 (12)	0.45355 (8)	0.0168 (2)	
C23	0.15472 (14)	0.65011 (13)	0.36161 (8)	0.0163 (2)	
H23	0.1308	0.7557	0.3537	0.020*	
C24	0.14290 (14)	0.55395 (13)	0.28038 (8)	0.0164 (2)	
C25	0.17807 (14)	0.40041 (13)	0.28998 (8)	0.0165 (2)	
H25	0.1656	0.3340	0.2339	0.020*	
C26	0.23134 (14)	0.34521 (12)	0.38199 (8)	0.0162 (2)	
O21	0.28898 (12)	0.38660 (9)	0.55580 (6)	0.02378 (19)	
C27	0.20933 (15)	0.70160 (13)	0.53734 (8)	0.0199 (2)	
O271	0.16416 (12)	0.83252 (9)	0.52573 (6)	0.02424 (19)	
O272	0.26860 (13)	0.65093 (10)	0.62536 (6)	0.0294 (2)	
H272	0.288 (2)	0.540 (2)	0.6144 (12)	0.044*	
N24	0.09824 (12)	0.61730 (11)	0.18421 (7)	0.01767 (19)	
O241	0.08439 (11)	0.75980 (9)	0.17920 (6)	0.02152 (18)	
O242	0.07873 (11)	0.52884 (10)	0.11242 (6)	0.02219 (18)	
N26	0.27842 (12)	0.18676 (11)	0.38689 (7)	0.01824 (19)	
O261	0.20718 (11)	0.09218 (9)	0.31673 (6)	0.02451 (19)	
O262	0.38998 (12)	0.15381 (10)	0.45879 (6)	0.02596 (19)	

### 4-(4-Nitrophenyl)piperazin-1-ium 2-carboxy-4,6-dinitrophenolate (II) Atomic displacement parameters (Å^2^)

**Table:**

	*U*^11^	*U*^22^	*U*^33^	*U*^12^	*U*^13^	*U*^23^
N11	0.0230 (5)	0.0128 (4)	0.0146 (4)	0.0040 (4)	0.0037 (4)	−0.0003 (4)
C12	0.0177 (5)	0.0181 (5)	0.0198 (5)	0.0037 (4)	0.0032 (4)	−0.0033 (4)
C13	0.0188 (5)	0.0191 (5)	0.0171 (5)	−0.0008 (4)	0.0051 (4)	−0.0030 (4)
N14	0.0184 (4)	0.0179 (4)	0.0142 (4)	0.0015 (4)	0.0045 (3)	−0.0018 (3)
C15	0.0195 (5)	0.0162 (5)	0.0159 (5)	0.0045 (4)	0.0048 (4)	−0.0012 (4)
C16	0.0194 (5)	0.0157 (5)	0.0175 (5)	0.0030 (4)	0.0066 (4)	−0.0006 (4)
C141	0.0210 (5)	0.0132 (5)	0.0128 (5)	0.0032 (4)	0.0044 (4)	0.0028 (4)
C142	0.0163 (5)	0.0207 (5)	0.0176 (5)	0.0029 (4)	0.0030 (4)	0.0004 (4)
C143	0.0192 (5)	0.0199 (5)	0.0173 (5)	0.0064 (4)	0.0063 (4)	0.0020 (4)
C144	0.0218 (5)	0.0130 (5)	0.0133 (5)	0.0031 (4)	0.0039 (4)	0.0005 (4)
C145	0.0175 (5)	0.0168 (5)	0.0170 (5)	0.0012 (4)	0.0045 (4)	0.0015 (4)
C146	0.0190 (5)	0.0176 (5)	0.0169 (5)	0.0031 (4)	0.0071 (4)	0.0011 (4)
N144	0.0211 (5)	0.0150 (4)	0.0161 (4)	0.0033 (4)	0.0054 (4)	0.0013 (3)
O141	0.0240 (4)	0.0273 (4)	0.0229 (4)	0.0058 (3)	0.0092 (3)	−0.0061 (3)
O142	0.0213 (4)	0.0234 (4)	0.0230 (4)	−0.0029 (3)	0.0058 (3)	−0.0057 (3)
C21	0.0188 (5)	0.0154 (5)	0.0162 (5)	0.0013 (4)	0.0039 (4)	−0.0003 (4)
C22	0.0183 (5)	0.0142 (5)	0.0172 (5)	0.0014 (4)	0.0042 (4)	−0.0017 (4)
C23	0.0156 (5)	0.0146 (5)	0.0187 (5)	0.0024 (4)	0.0043 (4)	−0.0001 (4)
C24	0.0151 (5)	0.0189 (5)	0.0146 (5)	0.0018 (4)	0.0034 (4)	0.0011 (4)
C25	0.0143 (5)	0.0176 (5)	0.0174 (5)	−0.0001 (4)	0.0054 (4)	−0.0042 (4)
C26	0.0164 (5)	0.0131 (5)	0.0196 (5)	0.0024 (4)	0.0059 (4)	−0.0013 (4)
O21	0.0394 (5)	0.0160 (4)	0.0151 (4)	0.0071 (3)	0.0044 (3)	0.0009 (3)
C27	0.0252 (6)	0.0158 (5)	0.0178 (5)	0.0013 (4)	0.0051 (4)	−0.0015 (4)
O271	0.0347 (5)	0.0153 (4)	0.0218 (4)	0.0066 (3)	0.0046 (3)	−0.0032 (3)
O272	0.0541 (6)	0.0178 (4)	0.0151 (4)	0.0096 (4)	0.0051 (4)	−0.0019 (3)
N24	0.0141 (4)	0.0220 (5)	0.0168 (4)	0.0022 (4)	0.0043 (3)	0.0004 (4)
O241	0.0228 (4)	0.0206 (4)	0.0216 (4)	0.0054 (3)	0.0056 (3)	0.0050 (3)
O242	0.0224 (4)	0.0284 (4)	0.0151 (4)	0.0022 (3)	0.0049 (3)	−0.0038 (3)
N26	0.0207 (5)	0.0153 (4)	0.0209 (5)	0.0031 (4)	0.0093 (4)	−0.0007 (4)
O261	0.0267 (4)	0.0178 (4)	0.0285 (4)	0.0023 (3)	0.0074 (3)	−0.0087 (3)
O262	0.0329 (5)	0.0245 (4)	0.0226 (4)	0.0132 (4)	0.0061 (3)	0.0031 (3)

### 4-(4-Nitrophenyl)piperazin-1-ium 2-carboxy-4,6-dinitrophenolate (II) Geometric parameters (Å, º)

**Table:**

N11—C16	1.4919 (14)	C145—C146	1.3765 (15)
N11—C12	1.4951 (14)	C145—H145	0.9500
N11—H11	0.894 (14)	C146—H146	0.9500
N11—H12	0.910 (14)	N144—O142	1.2315 (12)
C12—C13	1.5187 (15)	N144—O141	1.2355 (12)
C12—H12A	0.9900	C21—O21	1.2788 (13)
C12—H12B	0.9900	C21—C26	1.4340 (15)
C13—N14	1.4635 (14)	C21—C22	1.4394 (15)
C13—H13A	0.9900	C22—C23	1.3747 (15)
C13—H13B	0.9900	C22—C27	1.4844 (15)
N14—C141	1.3812 (13)	C23—C24	1.3869 (15)
N14—C15	1.4625 (14)	C23—H23	0.9500
C15—C16	1.5169 (15)	C24—C25	1.3811 (15)
C15—H15A	0.9900	C24—N24	1.4498 (13)
C15—H15B	0.9900	C25—C26	1.3750 (15)
C16—H16A	0.9900	C25—H25	0.9500
C16—H16B	0.9900	C26—N26	1.4540 (14)
C141—C142	1.4125 (15)	C27—O271	1.2236 (14)
C141—C146	1.4133 (15)	C27—O272	1.3171 (14)
C142—C143	1.3774 (15)	O272—H272	1.003 (18)
C142—H142	0.9500	N24—O242	1.2277 (12)
C143—C144	1.3881 (15)	N24—O241	1.2389 (12)
C143—H143	0.9500	N26—O262	1.2303 (12)
C144—C145	1.3875 (15)	N26—O261	1.2343 (12)
C144—N144	1.4437 (13)		
			
C16—N11—C12	113.90 (8)	C144—C143—H143	120.2
C16—N11—H11	108.4 (9)	C145—C144—C143	120.87 (10)
C12—N11—H11	108.3 (9)	C145—C144—N144	119.43 (10)
C16—N11—H12	111.0 (8)	C143—C144—N144	119.67 (10)
C12—N11—H12	108.3 (9)	C146—C145—C144	119.48 (10)
H11—N11—H12	106.8 (12)	C146—C145—H145	120.3
N11—C12—C13	110.16 (9)	C144—C145—H145	120.3
N11—C12—H12A	109.6	C145—C146—C141	121.42 (10)
C13—C12—H12A	109.6	C145—C146—H146	119.3
N11—C12—H12B	109.6	C141—C146—H146	119.3
C13—C12—H12B	109.6	O142—N144—O141	122.45 (9)
H12A—C12—H12B	108.1	O142—N144—C144	118.77 (9)
N14—C13—C12	109.99 (9)	O141—N144—C144	118.78 (9)
N14—C13—H13A	109.7	O21—C21—C26	124.86 (10)
C12—C13—H13A	109.7	O21—C21—C22	120.75 (9)
N14—C13—H13B	109.7	C26—C21—C22	114.38 (9)
C12—C13—H13B	109.7	C23—C22—C21	121.89 (10)
H13A—C13—H13B	108.2	C23—C22—C27	117.59 (10)
C141—N14—C15	121.02 (9)	C21—C22—C27	120.49 (10)
C141—N14—C13	121.10 (9)	C22—C23—C24	120.26 (10)
C15—N14—C13	109.06 (8)	C22—C23—H23	119.9
N14—C15—C16	110.28 (9)	C24—C23—H23	119.9
N14—C15—H15A	109.6	C25—C24—C23	120.99 (10)
C16—C15—H15A	109.6	C25—C24—N24	119.55 (9)
N14—C15—H15B	109.6	C23—C24—N24	119.43 (10)
C16—C15—H15B	109.6	C26—C25—C24	118.95 (10)
H15A—C15—H15B	108.1	C26—C25—H25	120.5
N11—C16—C15	109.87 (9)	C24—C25—H25	120.5
N11—C16—H16A	109.7	C25—C26—C21	123.45 (10)
C15—C16—H16A	109.7	C25—C26—N26	115.98 (9)
N11—C16—H16B	109.7	C21—C26—N26	120.55 (9)
C15—C16—H16B	109.7	O271—C27—O272	121.14 (10)
H16A—C16—H16B	108.2	O271—C27—C22	121.85 (10)
N14—C141—C142	121.47 (10)	O272—C27—C22	117.01 (10)
N14—C141—C146	121.20 (10)	C27—O272—H272	105.0 (10)
C142—C141—C146	117.30 (10)	O242—N24—O241	123.40 (9)
C143—C142—C141	121.27 (10)	O242—N24—C24	118.92 (9)
C143—C142—H142	119.4	O241—N24—C24	117.67 (9)
C141—C142—H142	119.4	O262—N26—O261	122.85 (9)
C142—C143—C144	119.59 (10)	O262—N26—C26	119.17 (9)
C142—C143—H143	120.2	O261—N26—C26	117.95 (9)
			
C16—N11—C12—C13	−50.40 (12)	C26—C21—C22—C23	−1.93 (15)
N11—C12—C13—N14	56.08 (11)	O21—C21—C22—C27	1.51 (16)
C12—C13—N14—C141	84.07 (12)	C26—C21—C22—C27	−179.98 (10)
C12—C13—N14—C15	−63.61 (11)	C21—C22—C23—C24	2.55 (16)
C141—N14—C15—C16	−83.73 (12)	C27—C22—C23—C24	−179.35 (10)
C13—N14—C15—C16	63.98 (11)	C22—C23—C24—C25	−0.39 (16)
C12—N11—C16—C15	50.41 (11)	C22—C23—C24—N24	−178.48 (9)
N14—C15—C16—N11	−56.47 (11)	C23—C24—C25—C26	−2.25 (16)
C15—N14—C141—C142	164.67 (10)	N24—C24—C25—C26	175.84 (9)
C13—N14—C141—C142	20.80 (15)	C24—C25—C26—C21	2.85 (16)
C15—N14—C141—C146	−17.23 (15)	C24—C25—C26—N26	−175.67 (9)
C13—N14—C141—C146	−161.10 (10)	O21—C21—C26—C25	177.64 (10)
N14—C141—C142—C143	175.90 (10)	C22—C21—C26—C25	−0.79 (16)
C146—C141—C142—C143	−2.27 (16)	O21—C21—C26—N26	−3.90 (17)
C141—C142—C143—C144	0.36 (16)	C22—C21—C26—N26	177.67 (9)
C142—C143—C144—C145	1.84 (16)	C23—C22—C27—O271	5.49 (17)
C142—C143—C144—N144	179.67 (10)	C21—C22—C27—O271	−176.38 (10)
C143—C144—C145—C146	−2.02 (16)	C23—C22—C27—O272	−174.21 (10)
N144—C144—C145—C146	−179.86 (10)	C21—C22—C27—O272	3.93 (16)
C144—C145—C146—C141	0.00 (16)	C25—C24—N24—O242	5.79 (14)
N14—C141—C146—C145	−176.08 (10)	C23—C24—N24—O242	−176.09 (9)
C142—C141—C146—C145	2.09 (16)	C25—C24—N24—O241	−173.14 (9)
C145—C144—N144—O142	1.91 (15)	C23—C24—N24—O241	4.98 (14)
C143—C144—N144—O142	−175.95 (10)	C25—C26—N26—O262	150.18 (10)
C145—C144—N144—O141	−177.77 (10)	C21—C26—N26—O262	−28.38 (15)
C143—C144—N144—O141	4.37 (15)	C25—C26—N26—O261	−27.86 (14)
O21—C21—C22—C23	179.56 (10)	C21—C26—N26—O261	153.57 (10)

### 4-(4-Nitrophenyl)piperazin-1-ium 2-carboxy-4,6-dinitrophenolate (II) Hydrogen-bond geometry (Å, º)

**Table:**

*D*—H···*A*	*D*—H	H···*A*	*D*···*A*	*D*—H···*A*
N11—H11···O21	0.894 (14)	1.869 (14)	2.7356 (12)	162.8 (13)
N11—H11···O262	0.894 (14)	2.396 (14)	2.8937 (13)	115.4 (11)
N11—H12···O271^i^	0.910 (14)	1.874 (14)	2.7668 (12)	166.2 (12)
O272—H272···O21	1.000 (17)	1.549 (17)	2.5020 (12)	157.4 (15)
C12—H12*B*···O142^ii^	0.99	2.41	3.3921 (14)	173
C16—H16*A*···O141^ii^	0.99	2.54	3.4906 (14)	161
C145—H145···O242^iii^	0.95	2.46	3.3927 (15)	168
C146—H146···O241^iv^	0.95	2.55	3.4227 (15)	153

Symmetry codes: (i) *x*, *y*−1, *z*; (ii) −*x*+1, −*y*+1, −*z*+2; (iii) *x*+1, *y*, *z*+1; (iv) −*x*+1, −*y*+1, −*z*+1.
